# Structural Brain Volumes of Individuals at Clinical High Risk for Psychosis: A Meta-analysis

**DOI:** 10.1016/j.bpsgos.2021.09.002

**Published:** 2021-09-30

**Authors:** Conrad E. Vissink, Inge Winter-van Rossum, Tyrone D. Cannon, Paolo Fusar-Poli, Rene S. Kahn, Matthijs G. Bossong

**Affiliations:** a Department of Psychiatry, University Medical Center Utrecht Brain Center, Utrecht University, Utrecht, The Netherlands; b Departments of Psychology and Psychiatry, Yale University, New Haven, Connecticut; c Department of Psychiatry, Icahn School of Medicine at Mount Sinai, New York, New York; d Early Psychosis: Interventions and Clinical-detection Laboratory, Department of Psychosis Studies, Institute of Psychiatry, Psychology & Neuroscience, King’s College London, London, United Kingdom; e OASIS Service, South London and Maudsley NHS Foundation Trust, London, United Kingdom; f National Institute for Health Research, Maudsley Biomedical Research Centre, South London and Maudsley NHS Foundation Trust, London, United Kingdom; g Department of Brain and Behavioral Sciences, University of Pavia, Pavia, Italy

**Keywords:** Brain volume, Clinical High Risk, Meta-analysis, Neuroimaging, Schizophrenia, Structural MRI

## Abstract

**Background:**

Structural magnetic resonance imaging studies in individuals at clinical high risk (CHR) for psychosis have yielded conflicting results.

**Methods:**

The aims of this study were to compare intracranial and structural brain volumes and variability of CHR individuals with those of healthy control (HC) subjects and to investigate brain volume differences and variability in CHR subjects with and without transition to psychosis. The PubMed and Embase databases were searched for relevant studies published before June 1, 2020.

**Results:**

A total of 34 studies were deemed eligible, which included baseline data of 2111 CHR and 1472 HC participants. In addition, data were included for 401 CHR subjects who subsequently transitioned to psychosis and 1023 nontransitioned CHR participants. Whole-brain and left, right, and bilateral hippocampal volume were significantly smaller in CHR subjects than in HC subjects. Cerebrospinal fluid and lateral ventricle volumes were significantly larger in CHR subjects than in HC subjects. Variability was not significantly different in CHR subjects compared with HC subjects. CHR individuals with and without subsequent transition to psychosis did not show significant differences in any of the volumetric assessments or in variability.

**Conclusions:**

This meta-analysis demonstrates reduced whole-brain and hippocampal volumes and increased cerebrospinal fluid and lateral ventricle volumes in CHR individuals. However, no significant differences were observed in any of the volumetric assessments between CHR individuals with and without subsequent transition to psychosis. These findings suggest that although structural brain alterations are present before the onset of the disorder, they may not significantly contribute to the identification of CHR individuals at the highest risk for the development of psychosis.

SEE COMMENTARY ON PAGE 90

Under standard treatment, psychotic disorders such as schizophrenia can display an impairing course characterized by functional and cognitive decline, as well as disturbances in perception and thought. Interventions at the time of a first psychotic episode can improve outcomes (1,2), but the subjective quality of life may already be compromised (3,4). The onset of the disorder is typically preceded by a clinical high-risk (CHR) state for psychosis, characterized by declined psychosocial functioning and subthreshold psychotic symptoms (5). Individuals in this stage have a 20% risk of developing psychosis in the following 2 years (6). Extensive research has focused on the pathophysiology of the disease, including an increasing amount of neuroimaging studies investigating volumetric changes in brain structures (7).

Extensive meta-analyses of neuroimaging evidence demonstrate that patients with schizophrenia exhibit significant morphometric brain volume abnormalities, such as smaller hippocampus and amygdala volumes and greater lateral ventricles (8,9). These abnormalities appear to be progressive (10) and may be partly explained by use of antipsychotic medication (11,12), although contradictory findings have been reported (13). These meta-analyses also show significantly reduced intracranial volume in patients with schizophrenia (8,9). Because intracranial volume directly reflects brain growth and the brain’s maximum size is thought to be reached around midadolescence (14), this indicates that smaller brain volume in schizophrenia is related to an early developmental process (15). Finally, it has been shown that schizophrenia is also associated with a significantly different variability of regional brain volumes, such as lower variability in anterior cingulate cortex and greater variability in temporal cortex and third ventricle volumes (16). Lower variability in the cingulate cortex suggests a biological effect of the disorder in this region because it is uniformly affected across all patients.

A substantial number of studies have focused on brain volumes in CHR individuals, thereby examining volume before the onset of the disorder and without potential confounding effects of antipsychotic medication, with contrasting results. For example, although initial studies demonstrated significant reductions in hippocampal volumes in CHR individuals compared with healthy control (HC) subjects (17,18), a meta-analysis focusing on hippocampal volume did not demonstrate structural brain differences between 939 CHR subjects and 490 HC subjects (19). A recent large multicenter study failed to show any significant brain and intracranial volume differences in 378 CHR individuals (20). Comparing brain volumes between 55 CHR individuals who subsequently made a transition to psychosis and 110 CHR individuals who did not develop psychosis, an exploratory meta-analysis by Smieskova *et al.* (21) suggested relatively larger intracranial and whole-brain volumes at baseline in CHR individuals with subsequent transition to psychosis. Voxel-based morphometry studies, investigating gray matter intensity on a voxel level rather than in predefined regions of interest, implicate widespread effects across all brain lobes and the cerebellum (22, 23, 24). However, it is currently still under debate whether CHR individuals exhibit intracranial volume, brain volume, and variability abnormalities, as has been shown in patients with schizophrenia (16).

Here, we present a meta-analysis focusing on intracranial and brain volumes of CHR individuals. The aims of the analysis were to compare structural brain volumes and interindividual variability in brain volumes 1) between individuals at CHR for psychosis and HC subjects and 2) between CHR individuals with subsequent transition to psychosis (CHR-T) and without subsequent transition to psychosis (CHR-NT).

## Methods and Materials

### Selection Procedures and Data Collection Search Strategy

Meta-analyses on reported brain volumes of CHR individuals were conducted following the Meta-analysis of Observational Studies in Epidemiology (MOOSE) and the Preferred Reporting Items for Systematic Reviews and Meta-analyses (PRISMA) guidelines (25,26). The PubMed and Embase databases were systematically searched for relevant studies published before June 1, 2020, using the following keywords: psychosis, neuroimaging or magnetic resonance imaging, and risk. All yielded articles were screened on title and abstract. Possible eligible papers were then full-text screened by 2 researchers (CEV and MGB) and selected after a consensus meeting. Reference lists were checked for other relevant publications.

### Eligibility Criteria

Original studies that compared structural brain volumes as measured with magnetic resonance imaging (MRI) 1) between CHR individuals and HC subjects and/or 2) between CHR-T and CHR-NT were included. The CHR status of participants had to be established based on validated instruments, such as the Comprehensive Assessment of At Risk Mental States, Structured Interview of Psychosis-risk Syndromes, or Brief Psychiatric Rating Scale. Because age and sex are significantly associated with brain volume variation (27), comparison groups (i.e., CHR vs. HC and CHR-T vs. CHR-NT) were at least matched on age and sex, and sufficient data should be available to calculate effect size and standard error. If the latter condition was not met, authors were requested to provide required data. In cases where one cohort was reported in multiple papers, data from the largest sample were included. Brain area volumes that were reported at least 3 times in literature were considered for this meta-analysis.

### Synthesis of Results

The following variables were recorded from each selected article: sample size, applied CHR criteria, mean age, sex, and mean and standard deviation (SD) of reported brain volumes. Pooled volumes were calculated when brain volumes were reported per hemisphere. Pooled means and SDs were calculated using the following formulas: pooled means = (N1 × M1 + N2 × M2)/(N1 + N2) and pooled SDs = √[(N1 − 1) × S1^2^ + (N2 − 1) × S2^2^]/[N1 + N2 − 2]). Variability of the reported brain volumes was determined as described previously (16). In short, variability was calculated by determining the natural logarithmic variability ratio (lnVR) for each study and each reported brain area, as follows: lnVR = ln(S1/S2) + (1/[2(N1 − 1)] − 1/[2(N2 − 1)]). Here, S1 and S2 are the SDs of each group (i.e., 1 = CHR or CHR-T; 2 = HC or CHR-NT), and n1 and n2 are the sample sizes for each group per study. To aid interpretation, lnVR values were back-transformed to the integer scale, and weighted mean VRs were reported for each brain area. Study quality and manuscript content was assessed with the Strengthening the Reporting of Observational Studies in Epidemiology (STROBE) scale (28), a checklist containing items that should be included in reports and manuscripts. Studies that scored below 15 points on the STROBE checklist were excluded.

### Statistical Analysis

Meta-analyses were conducted using the Comprehensive Meta-Analysis software, version 2.2.064 (2011) (Biostat Inc). Data were pooled using the random-effects model, and Hedges’ *g* effect sizes were calculated (29). Because the applied statistical method is of a conservative nature, we did not correct for multiple testing. Heterogeneity and publication bias were assessed using the *I*^*2*^ index and Egger’s test, respectively. An *I*^*2*^ of >75% was considered considerable inconsistency of findings across studies. Meta-regression was used to examine moderating effects of age and sex on brain volume. *p* < .05 was regarded as statistically significant.

## Results

### Study Characteristics

A total of 6476 abstracts were screened, of which 108 were assessed for eligibility. A total of 34 studies were included in the meta-analysis (Figure S1). The sample comprised 2111 CHR subjects, 1472 HC subjects, 401 CHR-T subjects, and 1023 CHR-NT subjects, as specified in Table 1. All included papers were deemed of sufficient quality based on the STROBE checklist. An overview of individual study characteristics is presented in Table S1.

**Table 1:** Overview of Selected Sample

	CHR Sample	HC Sample	CHR-T Sample	CHR-NT Sample
Number of Publications	32	32	17	17
Number of Participants	2111	1472	401	1023
Age, Years, Mean (SD)	21.6 (4.6)	22.7 (4.8)	21.4 (4.6)	21.3 (4.6)
Sex, Male, %	54.7%	55.2%	63%	54.9%

CHR, clinical high risk; CHR-NT, clinical high risk without subsequent psychosis; CHR-T, clinical high risk with subsequent psychosis; HC, healthy control.

### Brain Volume and Variability in CHR and HC Individuals

**Figure 1:**
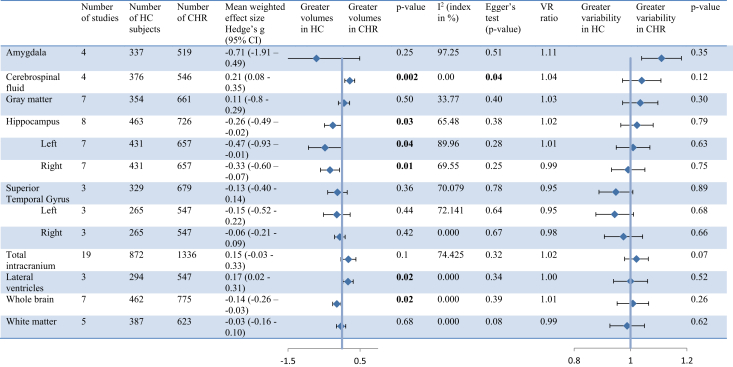
Brain volume and variability in clinical high risk (CHR) and healthy control (HC) individuals. CI, confidence interval; CSF, cerebrospinal fluid; VR, variability ratio.

Volumes of 13 brain structures were compared between CHR and HC individuals (Figure 1). Significant volume differences were found in six regions. Whole-brain and hippocampal volumes were significantly smaller in CHR individuals (Hedges’ *g* = −0.14, *p* = .02) than HC subjects (Hedges’ *g* = −0.26, *p* = .03). Hippocampal volume reduction was present in both the left (Hedges’ *g* = −0.47, *p* = .04) and right (Hedges’ *g* = −0.33, *p* = .01) hemispheres. Cerebrospinal fluid (CSF) volume and lateral ventricle volumes were significantly larger in CHR individuals (Hedges’ *g* = 0.21, *p* = .002) than HC subjects (Hedges’ *g* = 0.17, *p* = .02). Forest plots of individual areas are shown in Figures S2–S14. Variability in volume was not significantly different between CHR individuals and HC subjects for any of the brain regions (all *p* > .05) (Figure 1).

### Brain Volume and Variability in CHR-T and CHR-NT Individuals

Volume and variability of nine brain structures were compared between CHR-T and CHR-NT individuals (Figure 2). No significant differences were observed in any of the volumetric and variability assessments between CHR-T individuals and CHR-NT individuals (all *p* > .05). Forest plots of individual areas are shown in Figures S15–S23.

**Figure 2:**
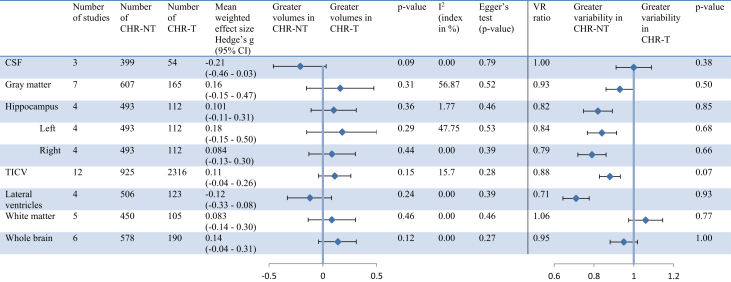
Brain volume and variability in clinical high risk with subsequent transition to psychosis (CHR-T) and clinical high risk without subsequent transition to psychosis (CHR-NT) individuals. CI, confidence interval; CSF; cerebrospinal fluid; TICV, total intracranial volume; VR, variability ratio.

### Publication Bias, Heterogeneity, and Meta-regression

When comparing brain volumes between CHR individuals and HC subjects, possible bias of selective publication was detected for studies reporting on CSF volumes (B0 = −1.92, *p* = .04). Considerable heterogeneity in reporting of left hippocampal and amygdala volumes was detected. There were no significant effects of heterogeneity. Meta-regression analysis found a small but significant moderating positive effect of age (estimate = 0.009, *p* = .02) and sex (estimate = 0.003, *p* = .02) on lateral ventricle volumes, indicating that older and male patients had relatively larger lateral ventricles. When comparing brain volumes between CHR-T and CHR-NT individuals, no significant effects of publication bias and heterogeneity were demonstrated. Meta-regression analysis yielded no significant moderating effects of age and sex.

## Discussion

This meta-analysis compiled brain volume data from 34 studies including 2111 CHR individuals and 1472 HC individuals. CHR subjects showed a significant volume reduction in whole-brain and hippocampus structures compared with HC subjects. In addition, the lateral ventricles and CSF were found to be significantly larger. There were no significant differences between CHR individuals and HC subjects in intracranial volume or brain volume variability. CHR subjects who transitioned to a first episode of psychosis did not differ on baseline volumetric and variability measures from CHR individuals without subsequent psychosis onset.

CHR individuals showed reduced whole-brain and hippocampal volumes and increased lateral ventricle and CSF volumes. Our finding of reduced hippocampal volume in the CHR group is consistent with results from a meta-analysis by Fusar-Poli *et al.* (24), which showed significant gray matter volume reductions in temporal regions including the hippocampus using a voxel-based morphometry approach. However, Walter *et al.* (19) performed a meta-analysis on eight studies comparing hippocampal volume of 1000 CHR subjects to almost 500 HC participants and did not find any significant differences. One possible explanation for the conflicting findings is that in contrast to the analyses performed by Walter *et al.*, in this meta-analysis, only structural MRI studies with CHR and HC groups matched on age and sex were included. Furthermore, our results are in line with extensive meta-analyses of structural brain alterations in patients with schizophrenia, which showed smaller whole-brain and hippocampal volumes and increased ventricle size and CSF volumes in patients compared with control subjects (8,30).

No brain volume differences were identified at baseline in our analysis between CHR-T individuals and CHR-NT individuals. In a previous meta-analysis, Smieskova *et al.* (21) demonstrated larger whole-brain volumes at baseline in CHR-T individuals compared with CHR-NT individuals. We were not able to confirm this, most likely because of our bigger sample size (190 CHR-T subjects and 578 CHR-NT subjects vs. 56 CHR-T subjects and 224 CHR-NT subjects). In addition, we did not find any significant effects of publication bias or heterogeneity, which emphasizes the robustness of our meta-analytic approach. Possible explanations for the absence of significant baseline differences in brain volumes between CHR-T and CHR-NT individuals could be found in the nature of the findings and the timing of assessments. First, although we did not find an association between baseline brain volumes and subsequent development of psychosis, machine learning studies were able to predict 84% and 88% of transitions in both genetic and CHR samples on the basis of whole-brain voxelwise neuroanatomical patterns (22,23). This indicates that baseline volumetric differences are present in other brain areas that are commonly investigated and/or are too subtle to detect in region of interest–based structural MRI studies. Second, longitudinal studies that examined structural brain volumes of CHR individuals at several time points showed that whole-brain volume was smaller and third ventricle volume increased faster over time in CHR-T individuals compared with CHR-NT individuals (31,32). This suggests that brain volume alterations related to psychosis onset may not be present at first clinical presentation but might occur later in the CHR phase or during psychosis onset. Third, NAPSL2 (North American Prodrome Longitudinal Study) found baseline anatomical differences associated with future transition among cases who were younger than 18 years at ascertainment, but not among those 18 years and older (20). Because the mean age of these study cohorts is 18 years or older in 31 of the 34 selected studies, the presented results may primarily reflect brain abnormalities in the adult rather than the mid- to late-adolescent population, which may show more pronounced neuroanatomical abnormalities. The final possibility is that the null hypothesis is true, namely that there are no structural alterations that can map the onset of psychosis in CHR individuals. This could be due to the high clinical and prognostic heterogeneity of CHR samples, mostly driven by idiosyncratic recruitment strategies and opportunistic sampling (33). It is also possible that structural imaging biomarkers are not suited to capture the subtle, dynamic neurobiological changes that are associated with onset of psychosis. This would align with the lack of validated structural biomarkers for established psychosis, despite several decades of research (34). Altogether, our results indicate that although baseline brain volumes are different between CHR and HC individuals, they do not distinguish CHR individuals from CHR-NT individuals. This further suggests that alterations in regional brain volumes may be related to the accumulation of nonpsychotic comorbid mental disorders in CHR samples rather than vulnerability to psychosis (35).

Elaborate meta-analyses showed significantly reduced intracranial volume in patients with schizophrenia (4,5). Because smaller intracranial volume indicates abnormal early brain development, it could be an important and highly reliable predictor for psychosis onset (15). However, we did not demonstrate intracranial volume differences between CHR and HC groups or between CHR-T and CHR-NT groups. Because intracranial volume is expected to be smaller in patients transitioned to psychosis, absence of intracranial volume differences between CHR-T and CHR-NT subjects may suggest that intracranial volume is affected in CHR-NT patients as well. Impaired intracranial volume development might be, rather than solely an indicator for schizophrenia, involved in CHR psychopathology. Many nontransitioned CHR patients will remain psychosocially impaired, and 50% to 70% have comorbid mental disorders (36).

To the best of our knowledge, we are the first to explore variability in brain volumes in the CHR population. No significant differences in brain volume variability between CHR and HC individuals or between CHR-T and CHR-NT individuals were identified in our analyses. This is in contrast with results from a meta-analysis on brain volume variability in patients with schizophrenia, which showed significant alterations in patients, such as lower variability in anterior cingulate cortex volume and greater variability in temporal cortex and third ventricle volumes (16). A finding with a significantly different variation compared with a control group may point out a biologically driven process and involvement with the pathophysiology of the disorder. The absence of variation differences indicate that no specific biological processes were detected and that these measures, although possibly significantly different, are varying too much for it to be indicated as part of a biologically driven process.

One possible explanation for the lack of alterations in brain volume variability in the CHR population could be the heterogeneity of the CHR concept. CHR individuals meet one or more of the following criteria: 1) attenuated psychotic symptoms; 2) brief, limited intermittent psychotic symptoms (BLIPS) (a history of 1 or more episodes of frank psychotic symptoms that resolved spontaneously within 1 week in the past year); or 3) a recent decline in function, together with either the presence of schizotypal personality disorder or a family history of psychosis in a first-degree relative. Although all subjects met CHR criteria, psychosis onset risk varies across categories, from 8% in genetic risk subjects to 38% in patients with BLIPS over 48-month follow-up (37). In addition, comorbid psychopathology such as anxiety and depression is common in the CHR population and could introduce variance in brain volume (38). Therefore, variability of brain volumes in the CHR group may not be different than that in the general population.

Several limitations should be taken into account. First, follow-up times varied substantially between studies included in this meta-analysis, ranging from a few months to multiple years. Because the risk of transition increases linearly from 10% in 6 months to 22% after 3 years (37), we cannot rule out that the CHR-NT group includes individuals that developed psychosis after the follow-up assessments were completed. Second, next to psychiatric comorbidities, multiple other confounding factors potentially affecting brain volume could not be taken into account, such as smoking, cannabis use, and cognitive functioning (39,40). Third, structural neuroimaging data from 19 papers were not included in this meta-analysis because cohorts were not matched on age and sex. However, because age and sex are significantly associated with brain volume variation (27) and the meta-analytic approach cannot take into account within-study cohort variation, this procedure strengthens interpretation of the results. Fourth, although subcortical structures such as the basal ganglia, cerebellum, and thalamus have been of great interest in the pathophysiology of both psychosis and in general CHR populations, unfortunately, insufficient data were available to include these regions.

Future studies should address the heterogeneity of the CHR population by examining the CHR designated subgroups (e.g., BLIPS) separately to reduce variability. An initiative, such as the clinical high-risk workgroup of ENIGMA (Enhancing Neuro Imaging Genetics through Meta Analysis), to merge available CHR MRI data from the scientific community presents a promising endeavor (41). In addition, an integration of imaging with other modalities, such as electroencephalography and blood biomarkers, may substantially improve psychosis prediction (42).

In conclusion, CHR individuals showed reduced whole-brain and hippocampal volumes and increased CSF and lateral ventricle volumes, but no intracranial volume abnormalities. CHR subjects who progressed to a first episode of psychosis did not differ on volumetric and variability measures from CHR individuals without subsequent psychosis onset. These findings suggest that although structural brain alterations are present before the onset of the disorder, they may not significantly contribute to the identification of CHR individuals at the highest risk for the development of psychosis.

## Supplementary Material

Supplementary Materials

Key Resources Table
