# Supplementary material for: Structural Brain Volumes of Individuals at Clinical High Risk for Psychosis: A Meta-analysis
Source: Biol Psychiatry Glob Open Sci. 2021 Sep 30;2(2):147–52. doi: 10.1016/j.bpsgos.2021.09.002 (PMC9616363; doi:10.1016/j.bpsgos.2021.09.002)
Supplement: Supplementary Materials [file mmc1.pdf]

Structural Brain Volumes of Individuals at Clinical High Risk for Psychosis: A Meta-analysis

Supplementary Information

Table S1 - Characteristics in individual study samples

Figure S2 - Forest plot of effect sizes of studies reporting on amygdala in CHR and HC subjects

Figure S3 - Forest plot of effect sizes of studies reporting on cerebrospinal fluid volume in CHR and HC subjects

Figure S4 - Forest plot of effect sizes of studies reporting on gray matter volume in CHR and HC subjects

Figure S5 - Forest plot of effect sizes of studies reporting on hippocampus volume in CHR and HC subjects

Figure S6 - Forest plot of effect sizes of studies reporting on left hippocampus volume in CHR and HC subjects

Figure S7 - Forest plot of effect sizes of studies reporting on right hippocampus volume in CHR and HC subjects

Figure S8 - Forest plot of effect sizes of studies reporting on superior temporal gyrus volume in CHR and HC subjects

Figure S9 - Forest plot of effect sizes of studies reporting on left superior temporal gyrus volume in CHR and HC subjects

Figure S10 - Forest plot of effect sizes of studies reporting on right superior temporal gyrus volume in CHR and HC subjects

Figure S11 - Forest plot of effect sizes of studies reporting on total intracranial volume in CHR and HC subjects

Figure S12 - Forest plot of effect sizes of studies reporting on lateral ventricles volume in CHR and HC subjects

Figure S13 - Forest plot of effect sizes of studies reporting on whole brain volume in CHR and HC subjects

Figure S14 - Forest plot of effect sizes of studies reporting on white brain volume in CHR and HC subjects

Figure S15 - Forest plot of effect sizes of studies reporting on cerebrospinal fluid volume in CHR-NT and CHR-T subjects

Figure S16 - Forest plot of effect sizes of studies reporting on gray matter volume in CHR-NT and CHR-T subjects

Figure S17 - Forest plot of effect sizes of studies reporting on hippocampus volume in CHR-NT and CHR-T subjects

Figure S18 - Forest plot of effect sizes of studies reporting on left hippocampus volume in CHR-NT and CHR-T subjects

Figure S19 - Forest plot of effect sizes of studies reporting on right hippocampus volume in CHR-NT and CHR-T subjects

Figure S20 - Forest plot of effect sizes of studies reporting on total intracranial volume in CHR-NT and CHR-T subjects

Figure S21 - Forest plot of effect sizes of studies reporting on lateral ventricles volume in CHR-NT and CHR-T subjects

Figure S22 - Forest plot of effect sizes of studies reporting on whole brain volume in CHR-NT and CHR-T subjects

Figure S23 - Forest plot of effect sizes of studies reporting on white matter volume in CHR-NT and CHR-T subjects

Supplemental References

Table S1. Studies included in the meta-analyses

Study		Study reference	N and diagnosis	Sex (% males)	Age (years ± SD)	Included brain regions	Quality score STROBE checklist
Amygdala subnucleus volumes in psychosis high-risk state and first episode psychosis	Armio et al, 2020	[1]	45 CHR<br>76 HC	56 %<br>43 %	25.8 ± 6.1<br>27.1 ± 4.9	CHR versus HC: amygdala, total intracranial volume	26
Regional Gray Matter Volume Abnormalities in the At Risk Mental State	Borgwardt et al, 2007	[2]	35 CHR<br>22 HC<br>12 CHR-T<br>23 CHR-NT	63 %<br>59 %<br>75 %<br>57 %	23.7 ± 5.6<br>23 ± 4.3<br>24.6 ± 5.3<br>23.3 ± 5.8	CHR versus HC: total intracranial volume<br><br>CHR-T versus CHR-NT: total intracranial volume	23
Hippocampus abnormalities in at risk mental states for psychosis? A cross-sectional high resolution region of interest magnetic resonance imaging study	Buehlmann et al, 2010	[3]	37 CHR<br>22 HC<br>16 CHR-T<br>21 CHR-NT	59 %<br>59 %<br>69 %<br>52 %	24.7 ± 5.6<br>23 ± 4.3<br>26.4 ± 6.5<br>23.4 ± 6	CHR versus HC: left hippocampus, right hippocampus, total hippocampus, whole brain<br><br>CHR-T versus CHR-NT: left hippocampus, right hippocampus, total hippocampus, whole brain	24
Cortical abnormalities in youth at clinical high-risk for psychosis: Findings from the NAPLS2 cohort	Chung et al, 2019	[4]	274 CHR<br>134 HC<br>35 CHR-T<br>239 CHR-NT	62 %<br>54 %<br>71 %<br>61 %	19.6 ± 4.2<br>20.5 ± 4.6<br>18.8 ± 3.8<br>19.7 ± 4.2	CHR versus HC: cerebrospinal fluid, gray matter, lateral ventricles, left hippocampus, right hippocampus, total hippocampus, left superior temporal gyrus, right superior temporal gyrus, total superior temporal gyrus, whole brain, total intracranial volume, white matter, amygdala<br><br>CHR-T versus CHR-NT: cerebrospinal fluid , gray matter, lateral ventricles, left hippocampus, right hippocampus, total hippocampus, total intracranial volume, whole brain, white matter	27
Disorganized Gyrification Network Properties During the Transition to Psychosis	Das et al, 2018	[5]	79 CHR<br>44 HC	72 %<br>39 %	24.3 ± 5.1<br>23 ± 4.3	CHR versus HC: total intracranial volume	22
Hippocampal Shape Abnormalities Predict Symptom Progression in Neuroleptic-Free Youth at Ultrahigh Risk for Psychosis	Dean et al, 2016	[6]	38 CHR<br>42 HC	50 %<br>58 %	18.9 ± 1.4<br>18.7 ± 1.9	CHR versus HC: left hippocampus, right hippocampus, total hippocampus,	27
Structural and functional alterations in the brain during working memory in medication-naive patients at clinical high-risk for psychosis	Gisselgard et al, 2018	[7]	41 CHR<br>37 HC	49 %<br>39 %	16.7 ± 2.4<br>16.9 ± 3	CHR versus HC: left hippocampus, right hippocampus, total hippocampus, total intracranial volume	24
Reduced volume in the anterior internal capsule but its maintained correlation with the frontal gray matter in subjects at ultra-high risk for psychosis	Han et al, 2012	[8]	43 CHR<br>42 HC	58%<br>62%	22.4 ± 4<br>22.8 ± 3.6	CHR versus HC: total intracranial volume	22

Caudate nucleus volume in individuals at ultra-high risk of psychosis: a cross-sectional magnetic resonance imaging study	Hannan et al, 2010	[9]	78 CHR<br>39 HC<br>39 CHR-T<br>19 CHR-NT	62%<br>62%<br>62%<br>65%	19.2 ± 3.3<br>20 ± 3.2<br>19.4 ± 3.3<br>19 ± 3.3	CHR versus HC: lateral ventricles, whole brain<br><br>CHR-T versus CHR-NT: lateral ventricles, whole brain, total intracranial volume	24
Interrelated neuropsychological and anatomical evidence of hippocampal pathology in the at-risk mental state	Hurlemann et al, 2008	[10]	36 CHR<br>30 HC	56 %<br>77 %	27.1 ± 5.6<br>28.2 ± 6.4	CHR versus HC: left hippocampus, right hippocampus, total hippocampus, whole brain	22
Localized gray matter volume reductions in the pars triangularis of the inferior frontal gyrus in individuals at clinical high-risk for psychosis and first episode for schizophrenia	Iwashiro et al, 2012	[11]	20 CHR<br>20 HC	50 %<br>50 %	21.4.x ± 3.6<br>22.6 ± 3.8	CHR versus HC: total intracranial volume	22
Cortical thickness reduction in individuals at ultra-high-risk for psychosis	Jung et al, 2011	[12]	29 CHR<br>29 HC	52 %<br>52 %	22.2 ± 4.3<br>23.2 ± 2.7	CHR versus HC: total intracranial volume	23
Symptom recovery and relationship to structure corpus callosum in individuals with an at risk mental state	Katagiri et al, 2018	[13]	37 CHR<br>16 HC	30 %<br>50 %	24.1 ± 7<br>23.2 ± 2.9	CHR versus HC: total intracranial volume	22
Lack of Evidence for Regional Brain Volume or Cortical Thickness Abnormalities in Youths at Clinical High Risk for Psychosis: Findings From the Longitudinal Youth at Risk Study	Klauser et al, 2015	[14]	69 CHR<br>32 HC	68 %<br>53 %	21.5 ± 3.5<br>23 ± 3.9	CHR versus HC: gray matter, bilateral hippocampus, total intracranial volume, white matter	24
Abnormal relationships between local and global brain measures in subjects at clinical high risk for psychosis: a pilot study	Konishi et al, 2018	[15]	19 CHR<br>20 HC	68%<br>60 %	20.9 ± 4.3<br>21.4 ± 3.7	CHR versus HC: amygdala	26
Neuroanatomical correlates of executive dysfunction in the at-risk mental state for psychosis	Koutselouris et al, 2010	[16]	40 CHR<br>30 HC<br>11 CHR-T<br>16 CHR-NT	68 %<br>60 %<br>82 %<br>67 %	24.5 ± 5.9<br>26.0 ± 2.7<br>21.6 ± 3.3<br>26.0 ± 6.8	CHR-T versus CHR-NT: total intracranial volume, white matter	26
Disease prediction in the at-risk mental state for psychosis using neuroanatomical biomarkers: results from the FePsy study	Koutselouris et al, 2012a	[17]	37 CHR<br>22 HC<br>16 CHR-T<br>21 CHR-NT	59%<br>59%<br>69%<br>52%	24.7 ± 6.2<br>23 ± 4.3<br>26.4 ± 6.5<br>23.4 ±6	CHR versus HC: Cerebrospinal fluid, total intracranial volume<br><br>CHR-T versus CHR-NT: cerebrospinal fluid, white matter, total intracranial volume	23
Multivariate patterns of brain-cognition associations relating to vulnerability and clinical outcome in the at-risk mental states for psychosis	Koutselouris et al, 2012b	[18]	40 CHR<br>30 HC<br>11 CHR-T<br>16 CHR-NT	68 %<br>60 %<br>82 %<br>69 %	24.5 ± 5.8<br>26 ± 2.7<br>21.6± 3.3<br>26 ± 6.8	CHR-T versus CHR-NT: grey matter	23
Use of neuroanatomical pattern classification to identify subjects in at-risk mental states of psychosis and predict disease transition	Koutsouleris et al, 2009	[19]	45 CHR<br>25 HC<br>15 CHR-T<br>18 CHR-NT	62 %<br>44 %<br>73 %<br>61 %	25.2 ± 5.9<br>25 ± 5.5<br>25.9 ± 6.7<br>22.4 ± 2.8	CHR versus HC: cerebrospinal fluid, gray matter, total intracranial volume, white matter<br><br>CHR-T versus CHR-NT: cerebrospinal fluid, gray matter, total intracranial volume, white matter	23

Neuroanatomical abnormalities that predate the onset of psychosis: a multicenter study	Mechelli et al, 2011	[20]	48 CHR-T<br>134 CHR-NT	31 %<br>38 %	22.7 ± 4.5<br>23.3 ± 5.3	CHR-T versus CHR-NT: gray matter	27
Structural brain alterations in subjects at high-risk of psychosis: a voxel-based morphometric study	Meisenzahl et al, 2008	[21]	40 CHR<br>75 HC	63 %<br>61 %	25 ± 5.6<br>25.1 ± 3.8	CHR versus HC: cerebrospinal fluid, total intracranial volume, white matter	22
Association of Adverse Outcomes With Emotion Processing and Its Neural Substrate in Individuals at Clinical High Risk for Psychosis	Modinos et al, 2020	[22]	213 CHR<br>52 HC<br>44 CHR-T<br>169 CHR-NT	51 %<br>52 %<br>57 %<br>49 %	22.9 ± 4.7<br>23.3 ± 4.0<br>22.6 ± 4.7<br>23.0 ± 4.7	CHR versus HC: total intracranial volume<br><br>CHR-T versus CHR-NT: total intracranial volume	25
Non-reduction in hippocampal volume is associated with higher risk of psychosis	Phillips et al, 2002	[23]	60 CHR<br>139 HC<br>20 CHR-T<br>40 CHR-NT	58 %<br>59 %<br>60 %<br>58 %	20.0 ± 3.3<br>30.1 ± 12.5<br>19.6 ± 3.7<br>20.2 ± 3.1	CHR-T versus CHR-NT: left hippocampus, right hippocampus, total hippocampus, whole brain	27
Subcortical Brain Volume Abnormalities in Individuals With an At-risk Mental State	Sabayashi et al, 2020	[24]	107 CHR<br>104 HC<br>21 CHR-T<br>72 CHR-NT	46 %<br>50 %<br>36 %<br>50 %	21.3 ± 5.4<br>22.6 ± 4.0<br>20.4 ± 4.4<br>21.7 ± 5.8	CHR-T versus CHR-NT: total intracranial volume, lateral ventricles, left hippocampus, right hippocampus, total hippocampus	24
Brain TSPO imaging and gray matter volume in schizophrenia patients and in people at ultra high risk of psychosis: An [11C]PBR28 study.	Selvaraj et al, 2018	[25]	14 CHR<br>14 HC	50 %<br>71 %	24.3 ± 5.4<br>28.1 ± 8	CHR versus HC: gray matter	26
Altered depth of the olfactory sulcus in ultra high-risk individuals and patients with psychotic disorders	Takahashi et al, 2014	[26]	135 CHR<br>87 HC<br>52 CHR-T<br>83 CHR-NT	58 %<br>63 %<br>58 %<br>58 %	20.1± 3.6<br>26.9 ± 10.1<br>19.6 ± 3.5<br>20.4 ± 3.6	CHR versus HC: whole brain<br><br>CHR-T versus CHR-NT: total intracranial volume, whole brain	24
Increased pituitary volume in subjects at risk for psychosis and patients with first-episode schizophrenia	Takahashi et al, 2013	[27]	22 CHR<br>22 HC	50 %<br>50 %	19.1 ± 4.1<br>19.4 ± 4.2	CHR versus HC:: total intracranial volume	24
Insular cortex gray matter changes in individuals at ultra-high-risk of developing psychosis	Takahashi et al, 2009a	[28]	97 CHR<br>55 HC<br>31 CHR-T<br>66 CHR-NT	61 %<br>65 %<br>65 %<br>59 %	19.8 ± 3.4<br>20.8 ± 3.6<br>19.1 ± 3.6<br>20.2 ± 3.3	CHR versus HC:: total intracranial volume<br><br>CHR-T versus CHR-NT: total intracranial volume	25
Progressive gray matter reduction of the superior temporal gyrus during transition to psychosis	Takahashi et al, 2009b	[29]	35 CHR<br>22 HC<br>12 CHR-T<br>23 CHR-NT	54 %<br>55 %<br>58 %<br>52 %	20 ± 4.4<br>22 ± 4.7<br>19.5 ± 5.1<br>20.2 ± 4	CHR versus HC: gray matter, left superior temporal gyrus, right superior temporal gyrus, total superior temporal gyrus, total intracranial volume<br><br>CHR-T versus CHR-NT: gray matter, total intracranial volume	25
Superior temporal gyrus volume in antipsychotic-naive people at risk of psychosis	Takahashi et al, 2010	[30]	97 CHR<br>42 HC	61 %<br>67 %	19.8 ± 3.4<br>20 ± 2.8	CHR versus HC:: left superior temporal gyrus, right superior temporal gyrus, total superior temporal gyrus	22
Hippocampal Subregions Across the Psychosis Spectrum	Vargas et al, 2018	[31]	61 CHR<br>70 HC	61 %<br>44 %	18.7 ± 1.8<br>18.3 ± 2.7	CHR versus HC:: left hippocampus, right hippocampus, total hippocampus, total intracranial volume	
Hippocampal subdivision and amygdalar volumes in	Witthaus et al, 2010	[32]	29 CHR<br>29 HC	66 %<br>59 %	25.3 ± 4.3<br>25.7 ± 5.2	CHR versus HC: amygdala, left	22

patients in an at-risk mental state for schizophrenia						hippocampus, right hippocampus, total hippocampus, whole brain	
No evidence for structural brain changes in young adolescents at ultra high risk for psychosis	Ziermans et al, 2009	[33]	54 CHR<br>54 HC	61 %<br>50 %	15.8 ± 2.1<br>15.8 ± 1.5	CHR versus HC: total intracranial volume, white matter, whole brain	26
Progressive structural brain changes during development of psychosis	Ziermans et al, 2012	[34]	43 CHR<br>30 HC<br>8 CHR-T<br>35 CHR-NT	67 %<br>50 %<br>88 %<br>63 %	15.6 ± 2.2<br>15.9 ± 1.4<br>16.8 ± 2.2<br>15.3 ± 2.1	CHR versus HC: gray matter, lateral ventricles<br><br>CHR-T versus CHR-NT: gray matter, lateral ventricles, total intracranial volume, whole brain, white matter	25

Note: CHR indicates Clinical high risk; HC, healthy control; CHR-T, Clinical high risk transitioned to psychosis, CHR-NT, Clinical high risk not transitioned to psychosis; SD, standard deviation.

Figure S1. PRISMA flow chart of manuscript selection

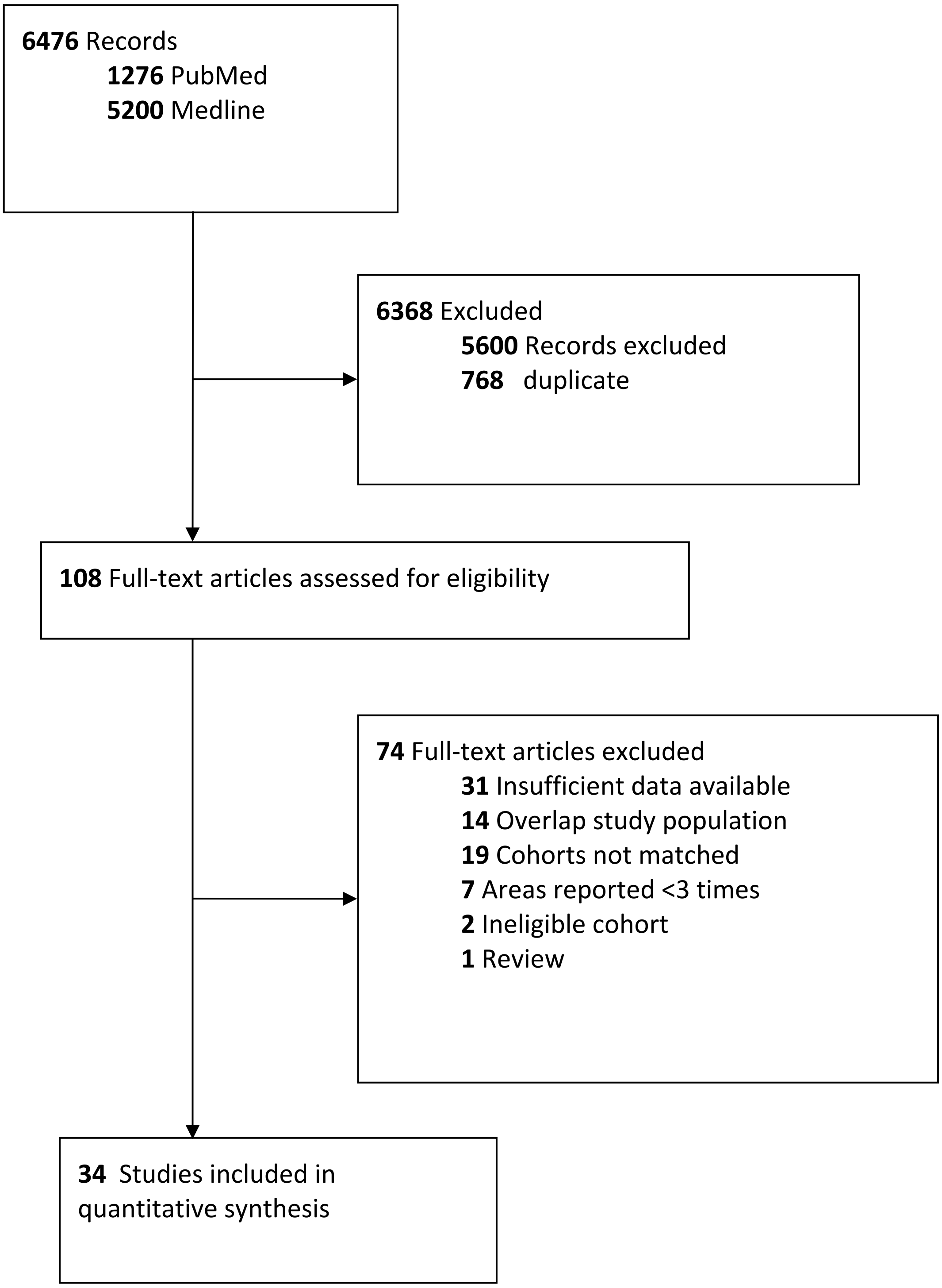

Figure S2 - Forest plot of effect sizes of studies reporting on amygdala in CHR and HC subjects

Study name	Hedges's g	Lower limit	Upper limit	p-Value	CHR	HC
Armio et al, 2020	-2.503	-2.945	-2.061	0.000	56	87
Chung et al, 2019	-0.023	-0.191	0.146	0.793	415	201
Konishi et al, 2018	-0.475	-1.099	0.149	0.136	19	20
Witthaus et al, 2010	0.157	-0.352	0.665	0.546	29	29
	-0.710	-1.907	0.487	0.245		

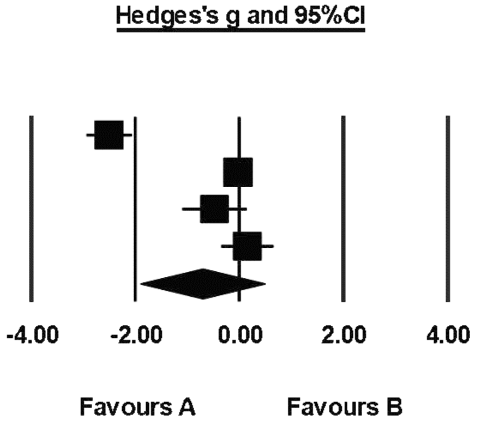

Figure S3 - Forest plot of effect sizes of studies reporting on cerebrospinal fluid volume in CHR and HC subjects

Study name	Hedges's g	Lower limit	Upper limit	p-Value	CHR	HC
Chung et al, 2019	0.296	0.127	0.465	0.001	415	201
Koutsouleris et al, 2009a	0.060	-0.424	0.544	0.808	45	25
Koutsouleris et al, 2012a	0.093	-0.272	0.458	0.616	46	75
Meisenzahl et al, 2008	0.021	-0.361	0.402	0.916	40	75
	0.214	0.077	0.350	0.002		

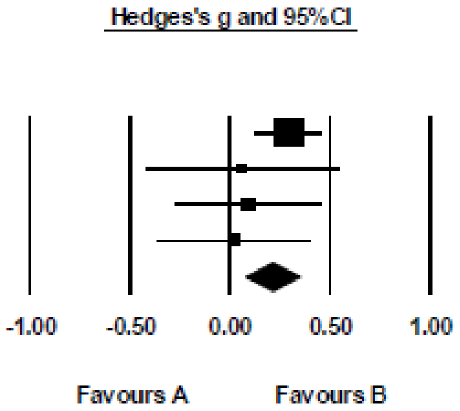

Figure S4 - Forest plot of effect sizes of studies reporting on gray matter volume in CHR and HC subjects

Study name	Hedges's g	Lower limit	Upper limit	p-Value	CHR	HC
Chung et al, 2019	-0.009	-0.177	0.159	0.918	415	201
Klauser et al, 2015	0.415	-0.005	0.835	0.053	69	32
Koutsouleris et al, 2009	0.259	-0.227	0.744	0.296	45	25
Koutsouleris et al, 2012b	0.453	-0.021	0.928	0.061	40	30
Selvaraj et al, 2018	-0.400	-1.126	0.327	0.281	14	14
Takahashi et al, 2009b	0.034	-0.492	0.560	0.898	35	22
Ziermans et al, 2012	-0.097	-0.558	0.365	0.682	43	30
	0.106	-0.080	0.292	0.264		

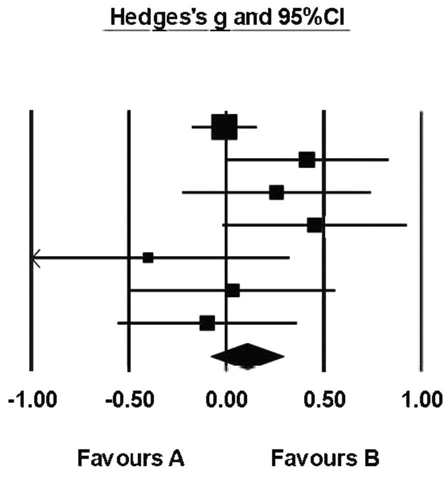

Figure S5 - Forest plot of effect sizes of studies reporting on hippocampus volume in CHR and HC subjects

Study name	Hedges's g	Lower limit	Upper limit	p-Value	CHR	HC
Buehlmann et al, 2010	0.153	-0.368	0.675	0.565	37	22
Chung et al, 2019	-0.117	-0.286	0.051	0.172	415	201
Dean et al, 2016	-0.495	-0.936	-0.054	0.028	38	42
Gisselgard et al, 2018	-0.467	-0.913	-0.021	0.040	41	37
Hurlemann et al, 2008	-1.142	-1.659	-0.625	0.000	36	30
Klauser et al, 2015	0.014	-0.402	0.430	0.948	69	32
Vargas et al, 2018	-0.068	-0.409	0.273	0.696	61	70
Witthaus et al, 2010	-0.095	-0.603	0.413	0.715	29	29
	-0.255	-0.488	-0.022	0.032		

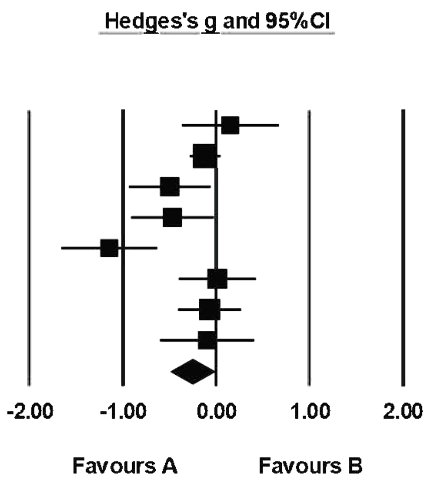

Figure S6 - Forest plot of effect sizes of studies reporting on left hippocampus volume in CHR and HC subjects

Study name	Hedges's g	Lower limit	Upper limit	p-Value	CHR	HC
Buehlmann et al, 2010	0.216	-0.306	0.738	0.417	37	22
Chung et al, 2019	-0.109	-0.278	0.059	0.203	415	201
Dean et al, 2016	-1.981	-2.513	-1.449	0.000	38	42
Gisselgard et al, 2018	-0.410	-0.855	0.034	0.071	41	37
Hurlemann et al, 2008	-1.107	-1.622	-0.592	0.000	36	30
Vargas et al, 2018	-0.052	-0.393	0.289	0.766	61	70
Witthaus et al, 2010	0.000	-0.508	0.508	1.000	29	29
	-0.473	-0.933	-0.013	0.044		

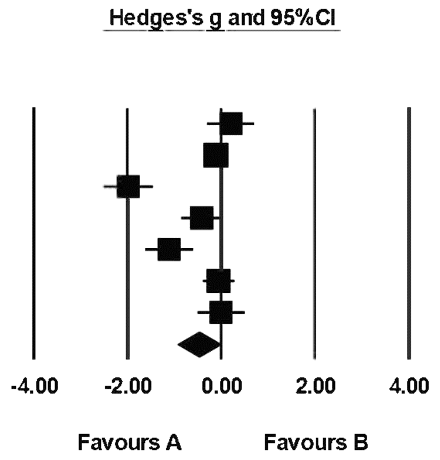

Figure S7 - Forest plot of effect sizes of studies reporting on right hippocampus volume in CHR and HC subjects

Study name	Hedges's g	Lower limit	Upper limit	p-Value	CHR	HC
Buehlmann et al, 2010	0.093	-0.428	0.614	0.726	37	22
Chung et al, 2019	-0.129	-0.298	0.039	0.132	415	201
Dean et al, 2016	-0.495	-0.936	-0.054	0.028	38	42
Gisselgard et al, 2018	-0.543	-0.991	-0.094	0.018	41	37
Hurlemann et al, 2008	-1.178	-1.698	-0.659	0.000	36	30
Vargas et al, 2018	-0.085	-0.427	0.256	0.624	61	70
Witthaus et al, 2010	-0.184	-0.693	0.325	0.479	29	29
	-0.334	-0.598	-0.070	0.013		

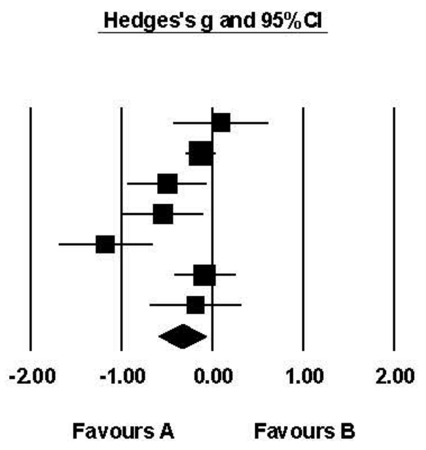

Figure S8 - Forest plot of effect sizes of studies reporting on superior temporal gyrus volume in CHR and HC subjects

Study name	Hedges's g	Lower limit	Upper limit	p-Value	UHR	HC
Chung et al, 2019	0.004	-0.164	0.173	0.958	415	201
Takahashi et al, 2009b	0.009	-0.365	0.384	0.962	70	44
Takahashi et al, 2010	-0.390	-0.647	-0.133	0.003	194	84
	-0.128	-0.399	0.143	0.356		

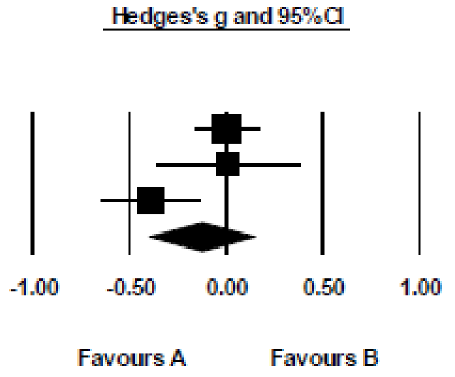

Figure S9 - Forest plot of effect sizes of studies reporting on left superior temporal gyrus volume in CHR and HC subjects

Study name	Hedges's g	Lower limit	Upper limit	p-Value	CHR	HC
Chung et al, 2019	0.033	-0.135	0.201	0.702	415	201
Takahashi et al, 2009b	0.027	-0.499	0.553	0.919	35	22
Takahashi et al, 2010	-0.513	-0.878	-0.148	0.006	97	42
	-0.146	-0.515	0.224	0.439		

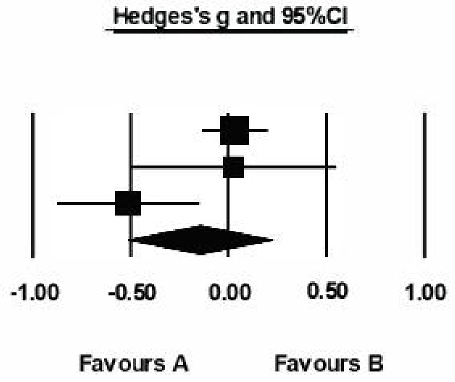

Figure S10 - Forest plot of effect sizes of studies reporting on right superior temporal gyrus volume in CHR and HC subjects

Study name	Hedges's g	Lower limit	Upper limit	p-Value	UHR	HC
Chung et al, 2019	-0.026	-0.195	0.142	0.758	415	201
Takahashi et al, 2009b	-0.013	-0.539	0.513	0.961	35	22
Takahashi et al, 2010	-0.240	-0.601	0.121	0.193	97	42
	-0.060	-0.207	0.086	0.418		

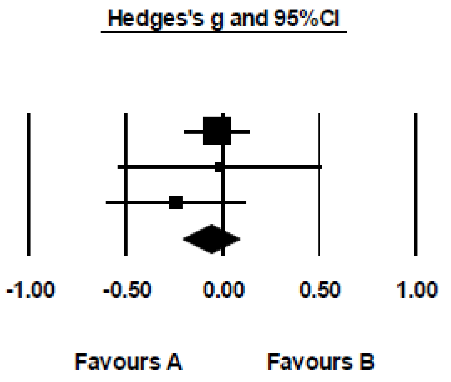

Figure S11 - Forest plot of effect sizes of studies reporting on total intracranial volume in CHR and HC subjects

Study name	Hedges's g	Lower limit	Upper limit	p-Value	CHR	HC
Armio et al, 2020	0.012	-0.322	0.346	0.946	56	87
Borgwardt et al, 2007	-0.045	-0.571	0.481	0.868	35	22
Chung et al, 2019	0.071	-0.097	0.240	0.406	415	201
Das et al, 2018	0.348	-0.021	0.717	0.065	79	44
Gisselgard et al, 2018	-0.464	-0.910	-0.018	0.041	41	37
Han et al, 2012	0.130	-0.292	0.552	0.546	43	42
Iwashiro et al, 2012	0.228	-0.382	0.837	0.464	20	20
Jung et al, 2011	0.341	-0.171	0.852	0.192	29	29
Katagiri et al, 2017	-0.053	-0.622	0.515	0.854	42	16
Klauser et al, 2015	0.374	-0.045	0.793	0.080	69	32
Koutsouleris et al, 2009a	0.169	-0.315	0.653	0.494	45	25
Koutsouleris et al, 2012a	-0.131	-0.653	0.390	0.622	37	22
Meisenzahl et al, 2008	0.158	-0.224	0.540	0.417	40	75
Modinos et al, 2020	0.001	-0.301	0.304	0.994	213	52
Takahashi et al, 2009a	-0.143	-0.472	0.187	0.396	97	55
Takahashi et al, 2009b	2.636	1.921	3.351	0.000	35	22
Takahashi et al, 2013	-0.282	-0.866	0.301	0.343	22	22
Vargas et al, 2018	0.451	0.106	0.797	0.010	61	70
Ziermans et al, 2009	-0.133	-0.508	0.242	0.486	54	54
	0.152	-0.029	0.333	0.100		

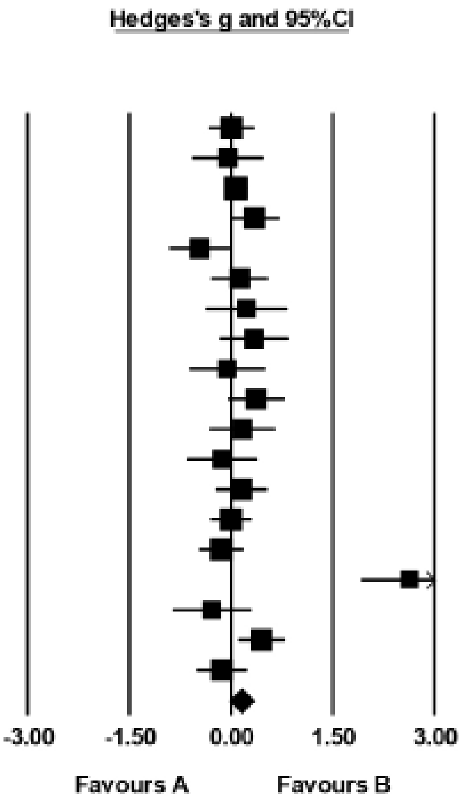

Figure S12 - Forest plot of effect sizes of studies reporting on lateral ventricles volume in CHR and HC subjects

Study name	Hedges's g	Lower limit	Upper limit	p-Value	CHR	HC
Chung et al, 2019	0.206	0.038	0.375	0.016	415	201
Hannan et al, 2010	-0.007	-0.389	0.374	0.969	78	39
Ziermans et al, 2009	0.138	-0.237	0.513	0.470	54	54
	0.167	0.024	0.309	0.022		

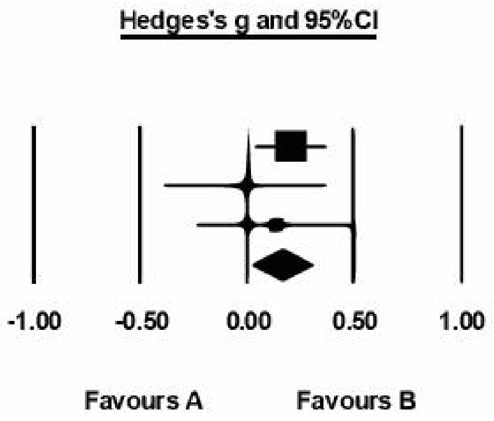

Figure S13 - Forest plot of effect sizes of studies reporting on whole brain volume in CHR and HC subjects

Study name	Statistics for each study					
	Hedges's g	Lower limit	Upper limit	p-Value	CHR	HC
Buehlman et al, 2010	-0.182	-0.704	0.340	0.495	37	22
Chung et al, 2019	-0.051	-0.219	0.117	0.551	415	201
Hannan et al, 2010	-0.338	-0.722	0.046	0.085	78	39
Hurlemann et al, 2008	-0.160	-0.678	0.359	0.546	26	30
Takahashi et al, 2014	-0.308	-0.578	-0.038	0.026	135	87
Witthaus et al, 2010	-0.032	-0.536	0.472	0.901	30	29
Ziermans et al, 2009	-0.138	-0.513	0.237	0.472	54	54
	-0.144	-0.260	-0.028	0.015		

Hedges's g and 95% CI

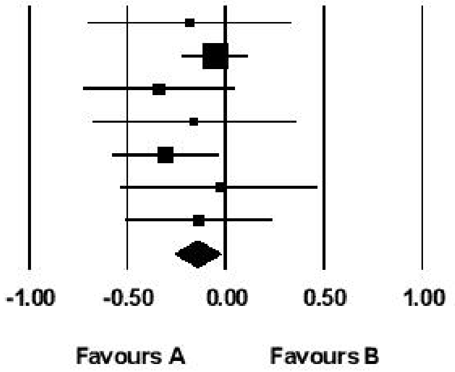

Figure S14 - Forest plot of effect sizes of studies reporting on white brain volume in CHR and HC subjects

Study name	Statistics for each study					
	Hedges's g	Lower limit	Upper limit	p-Value	CHR	HC
Chung et al, 2019	-0.092	-0.260	0.077	0.285	415	201
Klauser et al, 2015	0.196	-0.221	0.613	0.356	69	32
Koutsouleris et al, 2009a	0.099	-0.384	0.583	0.687	45	25
Meisenzahl et al, 2008	0.079	-0.302	0.460	0.685	40	75
Ziermans et al, 2009	-0.067	-0.442	0.307	0.724	54	54
	-0.027	-0.157	0.102	0.680		

Hedges's g and 95% CI

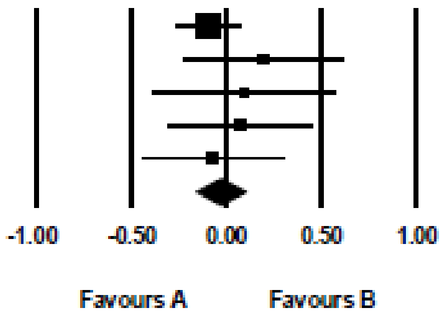

Figure S15 - Forest plot of effect sizes of studies reporting on cerebrospinal fluid volume in CHR-NT and CHR-T subjects

Study name	Statistics for each study				Sample size	
	Hedges's g	Lower limit	Upper limit	p-Value	CHR-T	CHR-NT
Chung et al, 2019	-0.236	-0.520	0.047	0.103	55	360
Koutsouleris et al, 2009a	-0.339	-1.013	0.334	0.323	15	18
Koutsouleris et al, 2012a	0.019	-0.618	0.655	0.954	16	21
	-0.213	-0.455	0.029	0.085		

Hedges's g and 95% CI

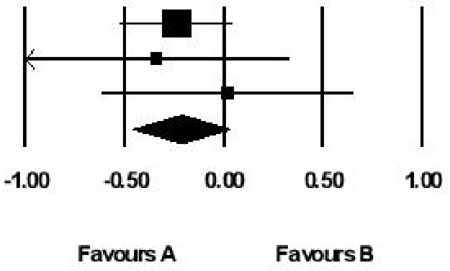

Figure S16 - Forest plot of effect sizes of studies reporting on gray matter volume in CHR-NT and CHR-T subjects

Study name	Hedges's g	Lower limit	Upper limit	p-Value	CHR-T	CHR-NT
	Statistics for each study				Sample size	
Chung et al, 2019	0.033	-0.250	0.316	0.819	55	360
Koutsouleris et al, 2009	-0.767	-1.461	-0.073	0.030	15	18
Koutsouleris et al, 2012a	0.003	-0.633	0.639	0.992	16	21
Koutsouleris et al, 2012b	0.945	0.159	1.731	0.018	11	16
Takahashi et al, 2009b	0.522	-0.171	1.215	0.140	12	23
Ziermans et al, 2012	0.617	-0.149	1.382	0.114	8	35
	0.188	-0.223	0.599	0.370		

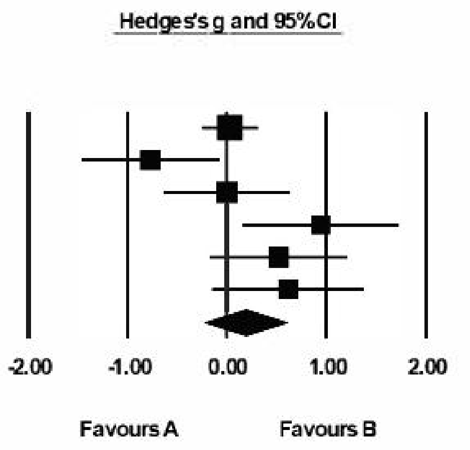

Figure S17 - Forest plot of effect sizes of studies reporting on hippocampus volume in CHR-NT and CHR-T subjects

Study name	Hedges's g	Lower limit	Upper limit	p-Value	CHR-T	CHR-NT
	Statistics for each study				Sample size	
Buehlmann et al, 2010	0.061	-0.576	0.697	0.851	16	21
Chung et al, 2019	0.004	-0.279	0.287	0.977	55	360
Phillips et al, 2002	0.538	-0.001	1.076	0.050	20	40
Sasabayashi et al, 2020	0.044	-0.438	0.526	0.859	21	72
	0.100	-0.112	0.313	0.355		

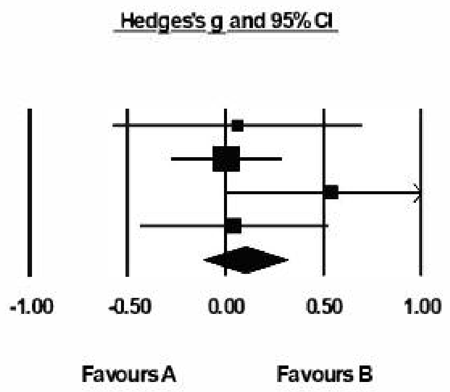

Figure S18 - Forest plot of effect sizes of studies reporting on left hippocampus volume in CHR-NT and CHR-T subjects

Study name	Hedges's g	Lower limit	Upper limit	p-Value	CHR-T	CHR-NT
	Statistics for each study				Sample size	
Buehlmann et al, 2010	0.107	-0.530	0.744	0.742	16	21
Chung et al, 2019	0.033	-0.250	0.317	0.818	55	360
Phillips et al, 2002	0.739	0.193	1.285	0.008	20	40
Sasabayashi et al, 2020	-0.043	-0.526	0.439	0.860	21	72
	0.175	-0.146	0.496	0.284		

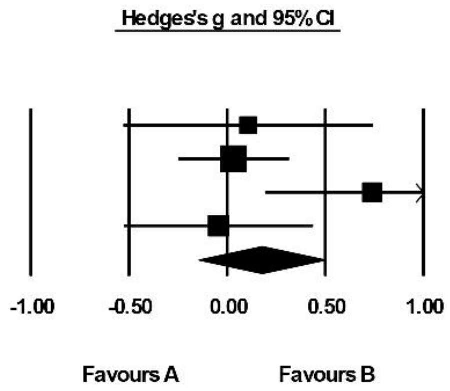

Figure S19 - Forest plot of effect sizes of studies reporting on right hippocampus volume in CHR-NT and CHR-T subjects

Study name	Statistics for each study				Sample size	
	Hedges's g	Lower limit	Upper limit	p-Value	CHR-T	CHR-NT
Buehlmann et al, 2010	0.015	-0.621	0.651	0.963	16	21
Chung et al, 2019	-0.002	-0.285	0.281	0.990	55	360
Phillips et al, 2002	0.356	-0.177	0.890	0.191	20	40
Sasabayashi et al, 2020	0.147	-0.335	0.630	0.550	21	72
	0.083	-0.126	0.293	0.436		

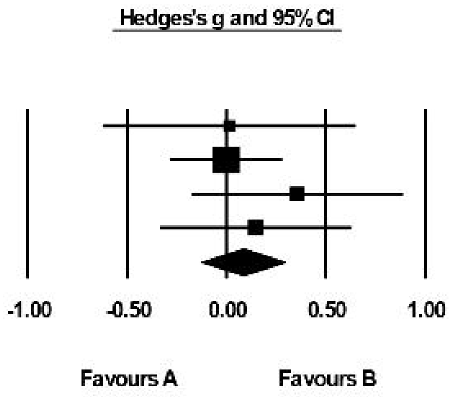

Figure S20 - Forest plot of effect sizes of studies reporting on total intracranial volume in CHR-NT and CHR-T subjects

Study name	Statistics for each study				Sample size	
	Hedges's g	Lower limit	Upper limit	p-Value	CHR-T	CHR-NT
Borgwardt et al, 2007	0.028	-0.654	0.710	0.936	12	23
Chung et al, 2019	0.064	-0.219	0.347	0.659	55	360
Hannan et al, 2010	0.269	-0.172	0.711	0.232	39	39
Koutsouleris et al, 2009	-0.510	-1.190	0.169	0.141	15	18
Koutsouleris et al, 2010	0.474	-0.282	1.229	0.219	11	16
Koutsouleris et al, 2012a	0.118	-0.519	0.755	0.716	16	21
Modinos et al, 2020	-0.137	-0.468	0.194	0.418	44	169
Sabayashi et al, 2020	-0.061	-0.543	0.421	0.805	21	72
Takahashi et al, 2009a	0.379	-0.047	0.806	0.081	31	66
Takahashi et al, 2009b	0.463	-0.228	1.153	0.189	12	23
Takahashi et al, 2014	0.074	-0.271	0.419	0.673	52	83
Ziermans et al, 2012	0.807	0.034	1.580	0.041	8	35
	0.110	-0.039	0.259	0.148		

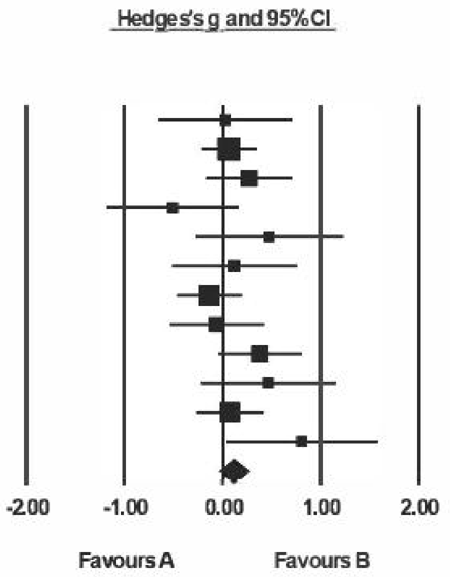

Figure S21 - Forest plot of effect sizes of studies reporting on lateral ventricles volume in CHR-NT and CHR-T subjects

Study name	Hedges's g	Lower limit	Upper limit	p-Value	CHR-T	CHR-NT
	Statistics for each study				Sample size	
Chung et al, 2019	-0.146	-0.429	0.138	0.313	55	360
Hannan et al, 2010	-0.079	-0.518	0.361	0.726	39	39
Sasabayashi et al, 2020	-0.241	-0.724	0.242	0.328	21	72
Ziermans et al, 2012	0.211	-0.544	0.966	0.584	8	35
	-0.122	-0.328	0.084	0.245		

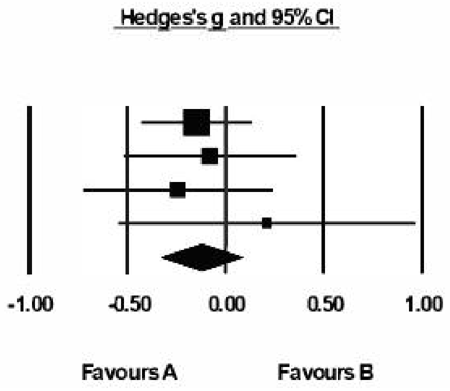

Figure S22 - Forest plot of effect sizes of studies reporting on whole brain volume in CHR-NT and CHR-T subjects

Study name	Hedges's g	Lower limit	Upper limit	p-Value	CHR-T	CHR-NT
	Statistics for each study				Sample size	
Buehlman et al, 2010	0.100	-0.537	0.737	0.758	16	21
Chung et al, 2019	0.086	-0.197	0.370	0.550	55	360
Hannan et al, 2010	0.328	-0.115	0.770	0.147	39	39
Phillips et al, 2002	0.000	-0.530	0.530	1.000	20	40
Takahashi et al, 2014	0.066	-0.279	0.410	0.709	52	83
Ziermans et al, 2012	0.625	-0.141	1.390	0.110	8	35
	0.137	-0.035	0.309	0.119		

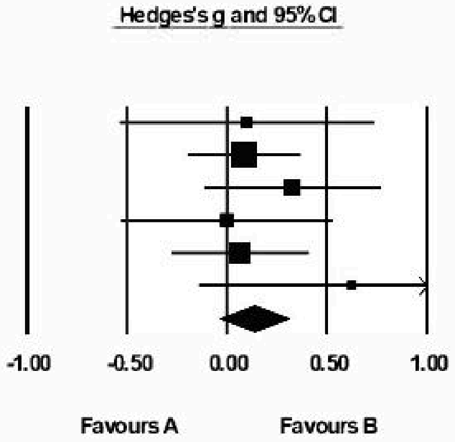

Figure S23 - Forest plot of effect sizes of studies reporting on white matter volume in CHR-NT and CHR-T subjects

Study name	Hedges's g	Lower limit	Upper limit	p-Value	CHR-T	CHR-NT
	Statistics for each study				Sample size	
Chung et al, 2019	0.045	-0.238	0.329	0.753	55	360
Koutsouleris et al, 2009	-0.060	-0.729	0.609	0.860	15	18
Koutsouleris et al, 2010	0.061	-0.684	0.806	0.872	11	16
Koutsouleris et al, 2012a	0.151	-0.486	0.789	0.642	16	21
Ziermans et al, 2012	0.459	-0.301	1.219	0.237	8	35
	0.083	-0.137	0.302	0.462		

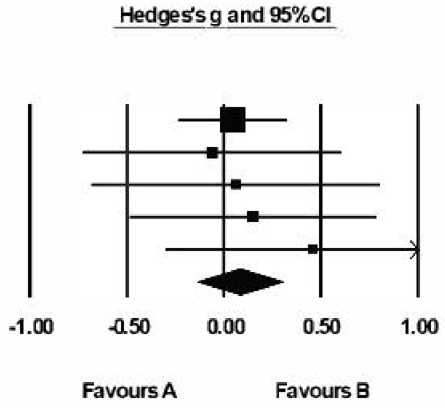

Supplemental References

1. Armio, R.-L., et al., *Amygdala subnucleus volumes in psychosis high-risk state and first-episode psychosis.* Schizophrenia Research, 2020. 215: p. 284-292.
2. Borgwardt, S.J., et al., *Regional gray matter volume abnormalities in the at risk mental state.* Biological psychiatry, 2007. 61(10): p. 1148-1156.
3. Buehlmann, E., et al., *Hippocampus abnormalities in at risk mental states for psychosis? A cross-sectional high resolution region of interest magnetic resonance imaging study.* Journal of psychiatric research, 2010. 44(7): p. 447-453.
4. Chung, Y., et al., *Cortical abnormalities in youth at clinical high-risk for psychosis: Findings from the NAPLS2 cohort.* NeuroImage: Clinical, 2019. 23: p. 101862.
5. Das, T., et al., *Disorganized gyrification network properties during the transition to psychosis.* Jama Psychiatry, 2018. 75(6): p. 613-622.
6. Dean, D.J., et al., *Hippocampal shape abnormalities predict symptom progression in neuroleptic-free youth at ultrahigh risk for psychosis.* Schizophrenia bulletin, 2016. 42(1): p. 161-169.
7. Gisselgard, J., et al., *Structural and functional alterations in the brain during working memory in medication-naive patients at clinical high-risk for psychosis.* PLoS One, 2018. 13(5): p. e0196289.
8. Han, H.J., et al., *Reduced volume in the anterior internal capsule but its maintained correlation with the frontal gray matter in subjects at ultra-high risk for psychosis.* Psychiatry Research: Neuroimaging, 2012. 204(2-3): p. 82-90.
9. Hannan, K.L., et al., *Caudate nucleus volume in individuals at ultra-high risk of psychosis: a cross-sectional magnetic resonance imaging study.* Psychiatry Research: Neuroimaging, 2010. 182(3): p. 223-230.
10. Hurlemann, R., et al., *Interrelated neuropsychological and anatomical evidence of hippocampal pathology in the at-risk mental state.* Psychological medicine, 2008. 38(6): p. 843-851.
11. Iwashiro, N., et al., *Localized gray matter volume reductions in the pars triangularis of the inferior frontal gyrus in individuals at clinical high-risk for psychosis and first episode for schizophrenia.* Schizophrenia research, 2012. 137(1-3): p. 124-131.
12. Jung, W.H., et al., *Cortical thickness reduction in individuals at ultra-high-risk for psychosis.* Schizophrenia bulletin, 2011. 37(4): p. 839-849.
13. Katagiri, N., et al., *Symptom recovery and relationship to structure of corpus callosum in individuals with an 'at risk mental state'.* Psychiatry Research: Neuroimaging, 2018. 272: p. 1-6.
14. Klauser, P., et al., *Lack of evidence for regional brain volume or cortical thickness abnormalities in youths at clinical high risk for psychosis: findings from the longitudinal youth at risk study.* Schizophrenia bulletin, 2015. 41(6): p. 1285-1293.
15. Konishi, J., et al., *Abnormal relationships between local and global brain measures in subjects at clinical high risk for psychosis: a pilot study.* Brain imaging and behavior, 2018. 12(4): p. 974-988.
16. Koutsouleris, N., et al., *Neuroanatomical correlates of executive dysfunction in the at-risk mental state for psychosis.* Schizophrenia research, 2010. 123(2-3): p. 160-174.
17. Koutsouleris, N., et al., *Disease prediction in the at-risk mental state for psychosis using neuroanatomical biomarkers: results from the FePsy study.* Schizophrenia bulletin, 2012. 38(6): p. 1234-1246.
18. Koutsouleris, N., et al., *Multivariate patterns of brain–cognition associations relating to vulnerability and clinical outcome in the at-risk mental states for psychosis.* Human brain mapping, 2012. 33(9): p. 2104-2124.
19. Koutsouleris, N., et al., *Use of neuroanatomical pattern classification to identify subjects in at-risk mental states of psychosis and predict disease transition.* Archives of general psychiatry, 2009. 66(7): p. 700-712.

20. Mechelli, A., et al., *Neuroanatomical abnormalities that predate the onset of psychosis: a multicenter study.* Archives of general psychiatry, 2011. 68(5): p. 489-495.
21. Meisenzahl, E., et al., *Structural brain alterations in subjects at high-risk of psychosis: a voxel-based morphometric study.* Schizophrenia research, 2008. 102(1-3): p. 150-162.
22. Modinos, G., et al., *Association of Adverse Outcomes With Emotion Processing and Its Neural Substrate in Individuals at Clinical High Risk for Psychosis.* JAMA psychiatry, 2020. 77(2): p. 190-200.
23. Phillips, L.J., et al., *Non-reduction in hippocampal volume is associated with higher risk of psychosis.* Schizophrenia research, 2002. 58(2-3): p. 145-158.
24. Sasabayashi, D., et al., *Subcortical brain volume abnormalities in individuals with an at-risk mental state.* Schizophrenia Bulletin, 2020.
25. Selvaraj, S., et al., *Brain TSPO imaging and gray matter volume in schizophrenia patients and in people at ultra high risk of psychosis: An [11C] PBR28 study.* Schizophrenia research, 2018. 195: p. 206-214.
26. Takahashi, T., et al., *Altered depth of the olfactory sulcus in ultra high-risk individuals and patients with psychotic disorders.* Schizophrenia research, 2014. 153(1-3): p. 18-24.
27. Takahashi, T., et al., *Increased pituitary volume in subjects at risk for psychosis and patients with first-episode schizophrenia.* Psychiatry and clinical neurosciences, 2013. 67(7): p. 540-548.
28. Takahashi, T., et al., *Insular cortex gray matter changes in individuals at ultra-high-risk of developing psychosis.* Schizophrenia research, 2009. 111(1-3): p. 94-102.
29. Takahashi, T., et al., *Progressive gray matter reduction of the superior temporal gyrus during transition to psychosis.* Archives of general psychiatry, 2009. 66(4): p. 366-376.
30. Takahashi, T., et al., *Superior temporal gyrus volume in antipsychotic-naive people at risk of psychosis.* The British Journal of Psychiatry, 2010. 196(3): p. 206-211.
31. Vargas, T., et al., *Hippocampal subregions across the psychosis spectrum.* Schizophrenia bulletin, 2018. 44(5): p. 1091-1099.
32. Witthaus, H., et al., *Hippocampal subdivision and amygdalar volumes in patients in an at-risk mental state for schizophrenia.* Journal of psychiatry & neuroscience: JPN, 2010. 35(1): p. 33.
33. Ziermans, T.B., et al., *No evidence for structural brain changes in young adolescents at ultra high risk for psychosis.* Schizophrenia research, 2009. 112(1-3): p. 1-6.
34. Ziermans, T.B., et al., *Progressive structural brain changes during development of psychosis.* Schizophr Bull, 2012. 38(3): p. 519-30.
